# Transplantation of umbilical cord-derived mesenchymal stem cells promotes the recovery of thin endometrium in rats

**DOI:** 10.1038/s41598-021-04454-7

**Published:** 2022-01-10

**Authors:** Lu Zhang, Ying Li, Yi-Chao Dong, Chun-Yi Guan, Shi Tian, Xiao-Dan Lv, Jian-Hui Li, Xing Su, Hong-Fei Xia, Xu Ma

**Affiliations:** 1 National Research Institute for Family Planning, Beijing, 100081 China; 2 Graduate School, Peking Union Medical College, Beijing, China; 3 Haidian Maternal and Child Health Hospital, Beijing, China

**Keywords:** Mesenchymal stem cells, Regeneration

## Abstract

The endometrium plays a critical role in embryo implantation and pregnancy, and a thin uterus is recognized as a key factor in embryo implantation failure. Umbilical cord mesenchymal stem cells (UC-MSCs) have attracted interest for the repair of intrauterine adhesions. The current study investigated the repair of thin endometrium in rats using the UC-MSCs and the mechanisms involved. Rats were injected with 95% ethanol to establish a model of thin endometrium. The rats were randomly divided into normal, sham, model, and UC-MSCs groups. Endometrial morphological alterations were observed by hematoxylin–eosin staining and Masson staining, and functional restoration was assessed by testing embryo implantation. The interaction between UC-MSCs and rat endometrial stromal cells (ESCs) was evaluated using a transwell 3D model and immunocytochemistry. Microarray mRNA and miRNA platforms were used for miRNA-mRNA expression profiling. Gene ontology (GO) and Kyoto encyclopedia of genes and genomes (KEGG) analyses were performed to identify the biological processes, molecular functions, cellular components, and pathways of endometrial injury and UC-MSCs transplantation repair and real-time quantitative reverse transcription PCR (qRT-PCR) was performed to further identify the expression changes of key molecules in the pathways. Endometrium thickness, number of glands, and the embryo implantation numbers were improved, and the degree of fibrosis was significantly alleviated by UC-MSCs treatment in the rat model of thin endometrium. In vitro cell experiments showed that UC-MSCs migrated to injured ESCs and enhanced their proliferation. miRNA microarray chip results showed that expression of 45 miRNAs was downregulated in the injured endometrium and upregulated after UC-MSCs transplantation. Likewise, expression of 39 miRNAs was upregulated in the injured endometrium and downregulated after UC-MSCs transplantation. The miRNA-mRNA interactions showed the changes in the miRNA and mRNA network during the processes of endometrial injury and repair. GO and KEGG analyses showed that the process of endometrial injury was mainly attributed to the decomposition of the extracellular matrix (ECM), protein degradation and absorption, and accompanying inflammation. The process of UC-MSCs transplantation and repair were accompanied by the reconstruction of the ECM, regulation of chemokines and inflammation, and cell proliferation and apoptosis. The key molecules involved in ECM-receptor interaction pathways were further verified by qRT-PCR. *Itga1* and *Thbs* expression decreased in the model group and increased by UC-MSCs transplantation, while *Laminin* and *Collagen* expression increased in both the model group and MSCs group, with greater expression observed in the latter. This study showed that UC-MSCs transplantation could promote recovery of thin endometrial morphology and function. Furthermore, it revealed the expression changes of miRNA and mRNA after endometrial injury and UC-MSCs transplantation repair processed, and signaling pathways that may be involved in endometrial injury and repair.

## Introduction

Normal embryos and healthy uteri are two necessary factors for a successful pregnancy^1^. The recent, continuous increase in induced abortions, uterine cavity operations, and the functional defects in endometrial stem/progenitor cells are believed to be responsible for thinning endometrium (< 7 mm)^2^. It has been reported that the thin endometrium is a defect of endometrial development and is associated with low embryo implantation rates^3–6^. In patients with a thin endometrium, it takes time to restore the endometrial thickness. Although various regimens such as estrogen therapy or low-dose aspirin therapy have been studied, effective treatment methods are lacking^1,7^. The anti-progestin mifepristone has been in use for more than 20 years as a medical alternative for early pregnancy termination^8^. Mifepristone alone may cause incomplete abortion, which is usually accompanied by uterine surgery. Mifepristone has been reported to inhibit the growth of ovarian cancer cells in vivo and in vitro as a cytostatic agent, and cause the damage and death of endometrial endothelial cells^9,10^.

Stem cell therapy has proven to be promising for the repair and regeneration of injured tissue. Human UC-MSCs have become the ideal choice for gene/cell therapy because of their several sources and thus easy availability, strong proliferation ability, and low immunogenicity among other superior properties. Mesenchymal stem cells (MSCs) sense the micro‐environment at the site of injury and secrete site-specific factors that play several important reparative roles in a multi-step process. First, MSCs migrate to the injured site to promote angiogenesis and revascularization by secreting cytokines, and accelerate wound healing^11,12^; second, MSCs promote the recovery of cellular functions at the injured site by exerting autocrine or paracrine effects^13^; third, MSCs secrete regulatory factors involved in the inflammatory response accompanying wound healing, thereby promoting injury repair^14^; lastly, MSCs secrete bioactive substances to inhibit scar formation and establish a regenerative microenvironment^15^. Previous studies have reported that bone marrow mesenchymal stem cells (BMSCs) transplantation increases the endometrial thickness and improves the injured uterus fertility^16,17^. An in-depth analysis of the tissue microenvironmental changes and elucidation of molecular mechanisms involved in endometrial injury and the UC-MSCs transplantation-mediated repair process will facilitate better reparative effects of UC-MSCs on the structure and function of the injured endometrium. The current study used a variety of methods to repair endometrial injury, though this was only partially accomplished and endometrial regeneration remains a significant challenge^18–20^.

Therefore, we evaluated the effectiveness of human UC-MSCs in a rat model of thin endometrial injury and on the recovery of impaired uterine function. We used a miRNA and mRNA chip platform to explore the differential characteristics of the endometrial microenvironment in injured endometrium and that subjected to UC-MSCs transplantation repair. By mapping the interaction network of miRNAs and mRNAs, as well as performing gene function annotation analysis and KEGG pathway analysis, the possible signaling pathways underlying tissue damage and repair processes were further explored.

## Materials and methods

### Experimental animals and groups

Six-weeks-old Sprague–Dawley (SD) mature rats were obtained from Hua Fu Kang Biotechnology Co., Ltd. [(Beijing, China) 2014-0004]. The rats were housed at room temperature (23–25 °C), with humidity between 40–60%, light/dark cycle 14 h/10 h, and free access to food and water.

Experimental animals were randomly divided into four groups, and 50 IU pregnant horse serum was injected to ensure synchronized physiological cycle. Rats were anesthetized with 2% pentobarbital sodium (40 mg/kg) and both sides of the "Y" type uterus were injected with 95% ethanol to establish a thin endometrial model. Rats in the normal group did not receive any treatment. In the sham group, PBS was injected into the uterus instead of 95% ethanol, and PBS was used instead of UC-MSCs during treatment. Rats in the model group were subjected to 95% ethanol-mediated endometrial injury and injected with the same volume of PBS through the tail vein eight days after surgery, as used for the UC-MSCs group. Rats in the UC-MSCs group were injected with 1 × 10^7^ cells/kg UC-MSCs suspension through the tail vein eight days after ethanol injury surgery. After two weeks, uteri were harvested from three animals of each group, and 50 IU pregnant horse serum was injected to ensure synchronized physiological cycle. Five rats were mated with sexually mature male rats (1:1 ratio), euthanized on the 9^th^ days by injection of an overdose pentobarbital sodium after the appearance of vaginal plugs. Uteri were harvested, and each uterus was examined to ascertain the number and location of fetuses.

### Compliance with ethical standards

The investigation was conducted according to the principles expressed in the Declaration of Helsinki. The study was approved by Ethics Committee of Research Institute for Family Planning and informed consent was obtained from all participants. The study was carried out in compliance with the ARRIVE guidelines.

### Isolation and characterization of UC-MSCs and ESCs

The human umbilical cords were obtained from full-term cesarean section surgeries performed at the Haidian Maternal and Child Health Hospital (Beijing, China). All patients provided written informed consent and the study was approved by the Ethics Committee of the National Research Institute for Family Planning. The tissue mass culture method was used to obtain UC-MSCs^21^. Briefly, the umbilical cord epithelium was removed, the cord vessels were separated, and the remaining tissue was cut into 1 mm^3^ pieces. Tissue pieces were tiled on 10-cm culture dishes, and the culture medium was added to the dishes. The tissues were cultured in α-MEM supplemented with 10% FBS and 1% penicillin–streptomycin medium and placed in a 37 °C 5% CO_2_ incubator until the cells grew around the tissue. F5 generation cells were used to analyze the expression of the surface antigens CD73, CD44, CD105, CD34, CD45, CD31 and HLA-DR, and the phenotype of UC-MSCs was assessed by fluorescence-activated cell sorting (FACS).

ESCs were isolated from the normal rat uteri^22^. The rat uterus was dissected longitudinally, and after several rounds of trypsin digestion, the cells were collected and filtered with a 600-mesh cell filter. ESCs were cultured in DMEM/F-12 medium supplemented with 10% FBS and 1% penicillin–streptomycin. After 3 days, the stromal cell-specific marker, vimentin was used to assess the purity of ESCs.

### Histological staining

Hematoxylin–eosin (H&E) staining was used to observe the tissue structure. The tissues were fixed with 4% paraformaldehyde overnight, dehydrated in a graded series of alcohol, embedded in paraffin, sliced (4 µm thick sections), deparaffinized, hydrated, and stained with hematoxylin and eosin. The morphological changes, endometrial thickness, and gland numbers were observed using a microscope (Nikon, Tokyo, Japan).

The endometrial thickness was analyzed quantitatively using length measurement software, and the number of glands was counted using the tissue slices. Five non-adjacent slices were randomly selected for each sample to record endometrial thickness, and three samples for each group were selected for statistical analysis. Five fields were randomly selected in each sample for gland counting, and three samples from each group were selected for statistical analysis.

Masson’s trichrome staining was performed to detect fibrosis. Briefly, the tissues were fixed with 4% paraformaldehyde overnight, dehydrated using a graded series of alcohol, paraffin embedded, sliced (4 µm thick sections), deparaffinized, hydrated, and subjected to Masson’s trichrome staining. A TE2000-U inverted phase contrast microscope (Nikon) was used to observe fibrosis in all samples. Five fields were randomly selected in each sample for analyzing fibrosis, and three samples from each group were selected for statistical analysis; ImageJ software was used to analyze the level of interstitial fibrosis in the endometrium.

### Immunofluorescence staining

Immunofluorescence was used to detect the expression of MAB1281. Rat uterus tissue was fixed with 4% paraformaldehyde overnight, and then dehydrated at 4 °C using 15% and 30% sucrose. After embedding the tissues at optimal cutting temperature, the tissues were cut into 10-µm slices. Slices were incubated with mouse anti-MAB1281 antibody (Millipore, USA) followed by TRITC-conjugated goat anti-mouse IgG, and the nuclei were stained with DAPI. The distribution of UC-MSCs was observed by fluorescence microscopy. Five fields of view were randomly selected for the analysis.

### MTT assay

Studies have shown that the concentration and the duration of mifepristone treatment can inhibit cell viability. To simulate the state of endometrial injury, the MTT assay was used to explore the effective concentration of mifepristone that affects ESC viability. ESCs were seeded in a 96-well plate and allowed to attach overnight. The cells were treated with a series of mifepristone medium (0, 10, 20, 50, 80, and 100 µM). After 48 h, the cells were incubated with 20 μL MTT (5 mg/ml) (Sigma-Aldrich, USA) at 37 °C for 2 h. Formazan crystals were dissolved in dimethyl sulfoxide (Sigma-Aldrich, USA) and the absorbance of each well was measured at 562 nm using SynergyMx microplate reader (BioTek). Samples were measured in triplicate, and all these assays were performed at least three times.

### Establishment of transwell 3D cultural model in vitro

A transwell chamber was used to establish a 3D culture model of ESCs and UC-MSCs. ESCs were seeded into a 24-well plate (60% proportion), allowed to adhere, and subsequently treated with 20 µM mifepristone for 12 h. The UC-MSCs suspension (5 × 10^6^/ml, 100 μL) was added to the upper chamber of the transwell system. The serum concentrations in the upper and lower transwell chambers were 1% and 5%, respectively. Twelve hours later, non-migrated UC-MSCs were removed from the upper chamber, while the migrated UC-MSCs at the bottom of the chamber membrane were fixed with 4% paraformaldehyde and stained with crystal violet. The number of migrated UC-MSCs was counted and the cell migration was analyzed by the number of cells passing through 8.0 µm chamber membrane.

The proliferation ability of the mifepristone-treated ESCs was analyzed by immunocytochemistry. Briefly, ESCs were fixed with 4% paraformaldehyde for 30 min, washed with PBS, and incubated with rabbit anti-Ki67 antibody (Abcam, Cambridge, UK; 1:200), followed by incubation with horseradish peroxidase (HRP)-conjugated goat anti-rabbit IgG (ZSJQ-Bio, Beijing, China, 1:100). The antibody binding was detected using diaminobenzidine (DAB), and the nuclei were stained with hematoxylin. The Ki-67 positive cells represented the proliferation of injured ESCs.

### mRNA and miRNA profiling

Total RNA was extracted from the uterus using Trizol reagent (Invitrogen, USA) according to the manufacturer’s instructions and the RNA samples were sent to LC Biotech (Hangzhou, China) for mRNA and miRNA microarray profiling. The differentially expressed genes were screened using the following standard: (1) fold change > 2 or < 0.5, (2) *p* value < 0.05, (3) false discovery rate (FDR)  < 0.05.

Principal component analysis (PCA) and unsupervised hierarchical clustering (HC) analysis were performed on differentially expressed miRNAs in three phases. GO and KEGG pathway analyses were performed using DAVID (https://david.ncifcrf.gov/summary.jsp) to identify the biological processes, molecular functions, cellular components, and pathways.

### Quantitative reverse-transcription polymerase chain reaction (qRT-PCR)

Microarray data was confirmed by qRT-PCR. Briefly, 1 μg of total RNA was reverse-transcribed using specific primers and a reverse transcription kit (Transgen Biotech, Beijing, China), Quantitative PCR was performed using FastStart Universal SYBR Green Master (Toyobo, Osaka, Japan) on a 7500 Real-Time PCR System (Applied Biosystems, USA). Cycling parameters were as follows: 95 °C for 10 min, and followed by 40 cycles of 95 °C 15 s and 60 °C 1 min. The primers for analyzing the differentially expressed genes and *Gapdh* were synthesized commercially and their sequences are listed in Table 1.

**Table 1:** Primer sequences.

Accession number	Primers	Sequences	Size (nt)	Tm (℃)	Annealing temperature (℃)
NM_030994	*Itga1*-qPCR-F	ACCAGTCAGCAGCTTCATTT	20	55	60
	*Itga1*-qPCR-R	CACAGTTCCGTTCCAGTCATAG	22	57	
NM_053889	*Vwf*-qPCR-F	TGGTCACACTCTCACATTTACTC	23	54	60
	*Vwf-*qPCR-R	CACAGATTCCACAGAGACCATAC	23	56	
NM_001169138	*Thbs*-qPCR-F	ACAGGCATAGGCTCGATTTATT	22	58	60
	*Thbs*-qPCR-R	CTGAACTCATGTGGAGTGAGAG	22	54	
NM_001108237	*Laminin*-qPCR-F	CATCGTTCCACGGGTGTATTA	21	58	60
	*Laminin*-qPCR-F	GCATGTGTCCAGCTCTACTT	20	52	
NM_053304	*Collagen*-qPCR-F-rat	GCTTGAAGACCTATGTGGGTATAA	24	58	60
	*Collagen*-qPCR-R-rat	GGGTGGAGAAAGGAACAGAAA	21	58	
NM_017008	*Gapdh*-qPCR-F	GCAAGGATACTGAGAGCAAGAG	22	56	60
	*Gapdh*-qPCR-R	GGATGGAATTGTGAGGGAGATG	22	61	

### Statistical analysis

All experimental data were expressed as the mean ± SEM. Experiments were performed at least three times. One-way ANOVA analysis was used for comparisons among different groups, and an independent sample *t*-test was used to compare the means between the two groups. Microarray intensity values were transformed into a log10 scale, and fold changes were calculated on a log2 scale. For the GO analysis, the *p* value was transformed into a log10 scale. Statistical significance was set at *p* < 0.05.

### Ethics approval

The animal-related experiments, including the isolation of UC-MSCs, the rat endometrial injury modeling, were approved by the Animal Care and Utilization Committee of National Research Institute for Family Planning (Ethics Number 2015-16). All applicable institutional and national guidelines for the care and use of animals were followed.

### Consent for publication

Our manuscript does not contain any individual person’s data in any form. All authors gave consent for publication.

## Results

### In vitro culture and phenotypic identification of UC-MSCs

The surface antigens of the F5 generation UC-MSCs were analyzed by flow cytometry. Over 95% UC-MSCs were positive for CD73, CD44, and CD105, and negative for CD34, CD45, CD31, and HLA-DR (Additional file 1: Figure S1a). UC-MSCs were fibroblast-like adherent cells and showed an orderly arrangement (Additional file 1: Figure S1b). Immunocytochemical results showed high expression of the CD44 surface antigen (Additional file 1: Figure S1c). Osteogenesis and adipogenesis were confirmed by alizarin red staining and oil red O staining, respectively (Additional file 1: Figure S1d–e).

### UC-MSCs ameliorate the appearance and morphological features of the thin uterus

**Figure 1:**
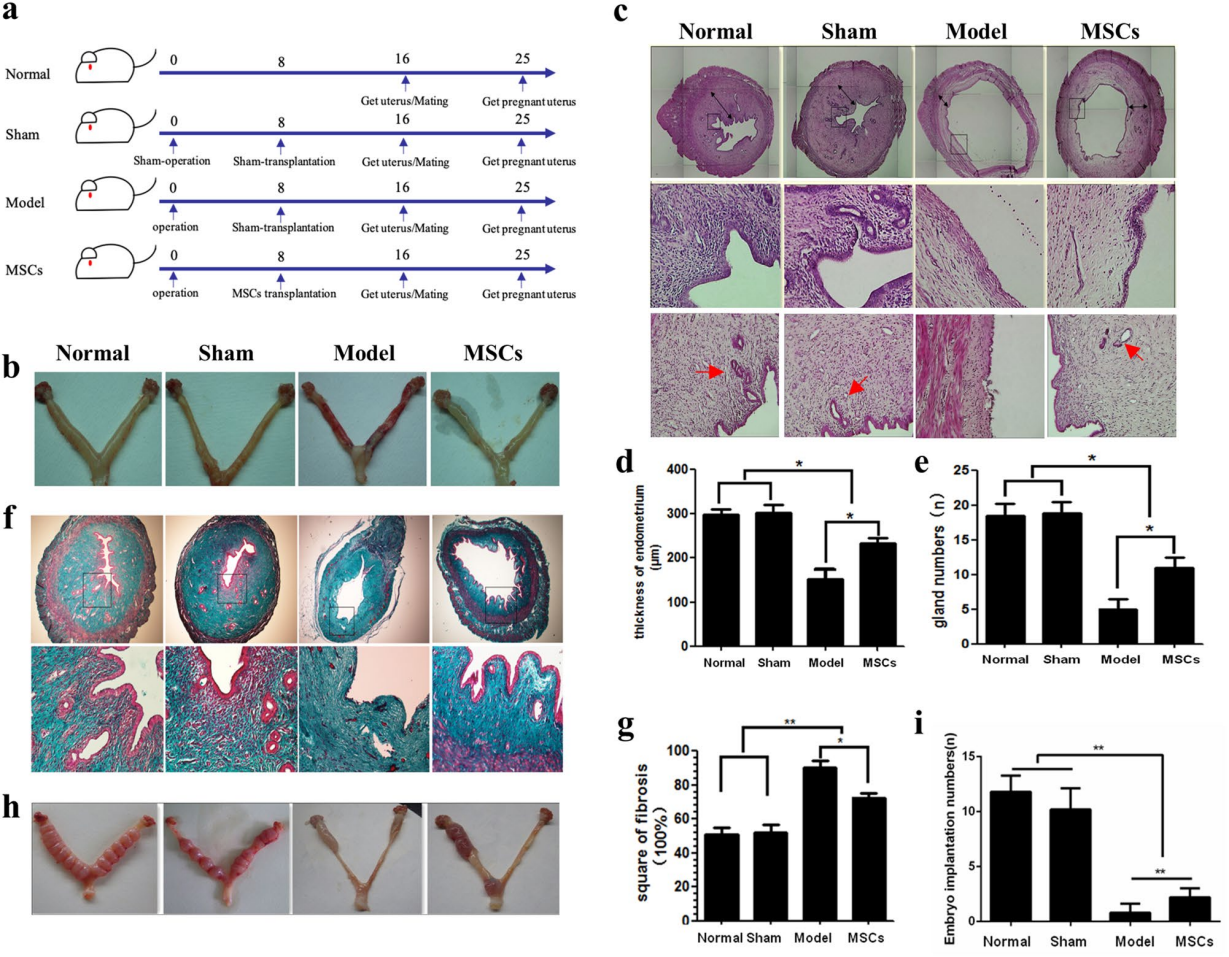
Uterine morphological and functional features and changes. (**a**) Experimental grouping schedule. (**b**) The specimen of uterus. (**c**) The H&E staining of rat uterine tissue (50× and 200×). The red arrow indicates the glands. (**d**) Statistical analysis of endometrial thickness. (**e**) Statistical analysis of gland numbers. (**f**) The Masson staining of rat uterine tissue (50× and 200×). (**g**) Statistical analysis of endometrial fibrosis. (**h**) The specimen of embryos implanted in the uterus. (**i**) Statistical analysis of embryos implanted numbers. *p < 0.05, **p < 0.01.

Experimental animals were divided into normal, sham, model and MSCs groups randomly (Fig. 1a). The uteri in the normal and sham group animals displayed uniform thickness and a smooth appearance. Comparatively, the uterus in the animals of the model group were rough, uneven in thickness, and showed marked hyperemia. UC-MSCs transplantation relieved the uterine congestion and edema, and the injury was partially repaired (Fig. 1b). H&E staining showed that the endometrial structure was relatively complete, and the epithelial cells were arranged closely in the normal group. Visible blood vessels and glands were observed. The uterus in the model group was significantly thin and the epithelium was missing (Fig. 1c).

Average endometrial thickness in the animals of the normal group was 298.7 ± 12.32 μm, whilst that of animals in sham group averaged 303.6 ± 18.05 μm. Epithelial cells in the uteri of model group animals were not closely arranged, and the average endometrial thickness was 153 ± 21.56 μm. The endometrial thickness in the MSCs group (233.2 ± 12.04 μm) was significantly lesser than that in the normal and sham groups yet significantly thicker than the model group (*p* < 0.05, Fig. 1d).

In the normal and sham groups, the glandular structures were intact, and the numbers were uniform. The number of glands was significantly reduced in the model group, and some glands were atrophied or incompletely formed. The endometrial glands in the MSCs group were sparse, and their number was significantly reduced; however, it was higher than that in the model group, and the structure was relatively complete (Fig. 1e).

Endometrial fibrosis is an indicator of the extent of uterine injury. Masson’s trichrome staining results showed that the intimal fibrosis area in the model group accounted for approximately 95% of the total endometrial area; however, this proportion was approximately 50% in the normal and sham groups, and 79% in the MSCs group. There was a significant difference between the model and MSCs groups, which suggested that UC-MSCs can repair the endometrium and inhibit the enhanced fibrosis of the interstitial fibrous connective tissue (Fig. 1f–g).

Observational and statistical analysis of uterine embryo implantation revealed that rats in both normal and sham group had a tighter arrangement of embryo implantation in the uterus, while those in the model group showed almost no embryo implantation. In the MSCs group, embryos were implanted in part of the uterus (Fig. 1h), and the number of embryos implanted was significantly lower compared to the normal and sham groups, but higher than model group (Fig. 1i).

### UC-MSCs migrated to the injured ESCs and enhance their proliferative capacity

**Figure 2:**
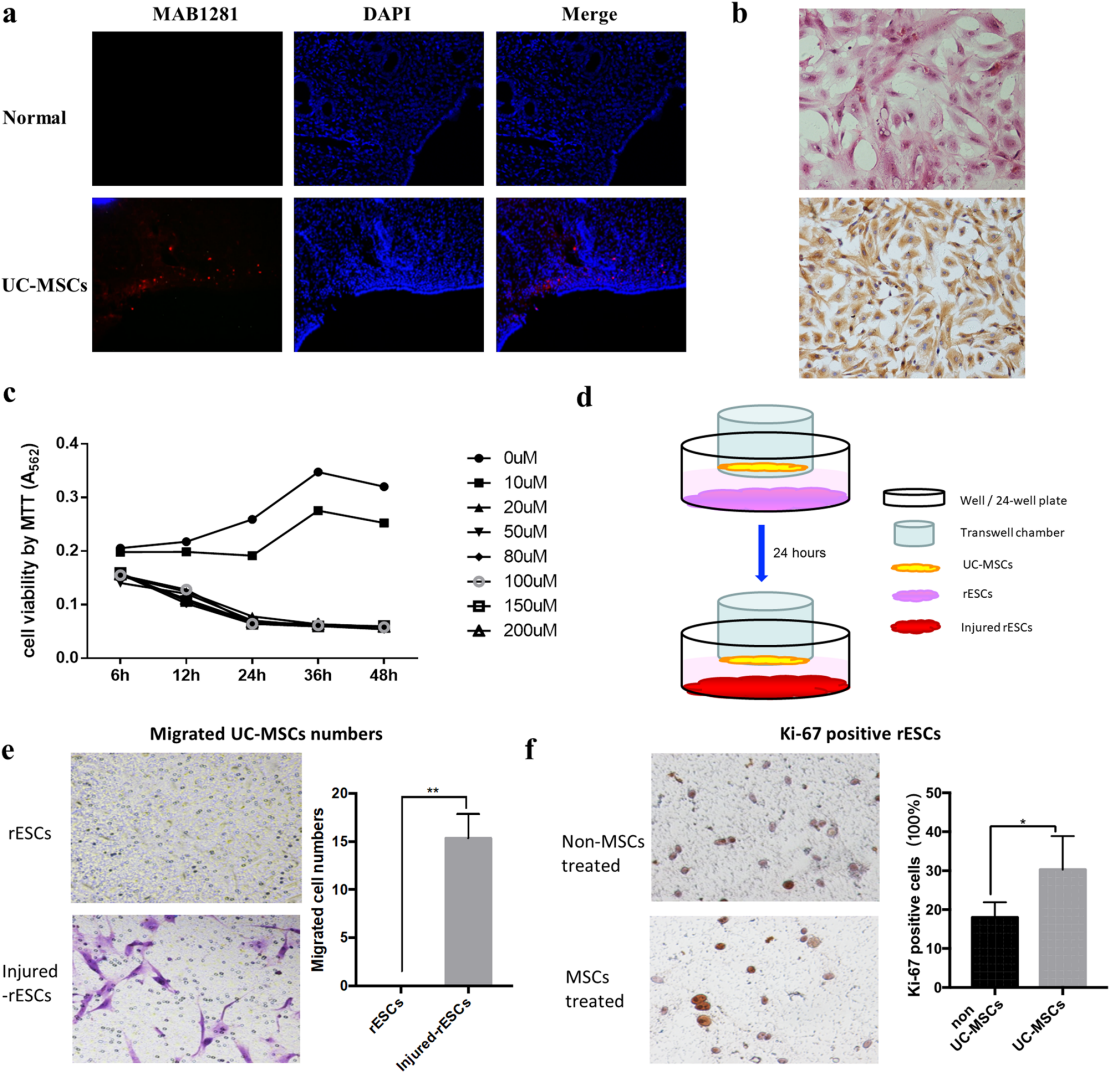
The migration and function of UC-MSCs. (**a**) UC-MSCs migrated to the injured endometrium. (**b**) The H&E staining and immunohistochemical of ESCs. (**c**) Viability of ESCs treated with mifepristone. (**d**) Schematic diagram of UC-MSCs-ESCs transwell 3D model. (**e**) The migration of UC-MSCs by transwell 3D mode. (**f**) The ESCs proliferation by transwell 3D mode. *p < 0.05, **p < 0.01.

MAB1281 immunofluorescence was assessed in the uteri of UC-MSCs transplantation rats and those of normal rats. The UC-MSCs group showed positive staining in the superficial layer of the uterus, indicating that UC-MSCs migrated to the damaged uterine part through the tail vein (Fig. 2a). ESCs culture was performed as described by McCormack and Glasser^22^. Results of the H&E and immunochemical analyses, performed to detect cell morphology and expression of stromal cell-specific marker vimentin, showed the high purity of the ESCs (Fig. 2b).

To better understand the function and reparative effects of UC-MSCs, an in vitro 3D UC-MSCs/ESCs culture model was established using a Transwell chamber. ESCs were treated by mifepristone to simulate endometrial injury in vitro. Our results showed that 20 µM mifepristone significantly reduced cell viability (Fig. 2c). As shown in Fig. 2d, the number of migrated UC-MSCs was significantly higher in the 20 µM mifepristone-treated group (20 µM -mife) compared with the no mifepristone group (0 µM -mife) (Fig. 2e). Immunochemistry showed that UC-MSCs treatment significantly increased the Ki-67 expression in mifepristone treated ESCs compared with the non-MSCs-treated group (Fig. 2f).

### miRNAs profiling

**Figure 3:**
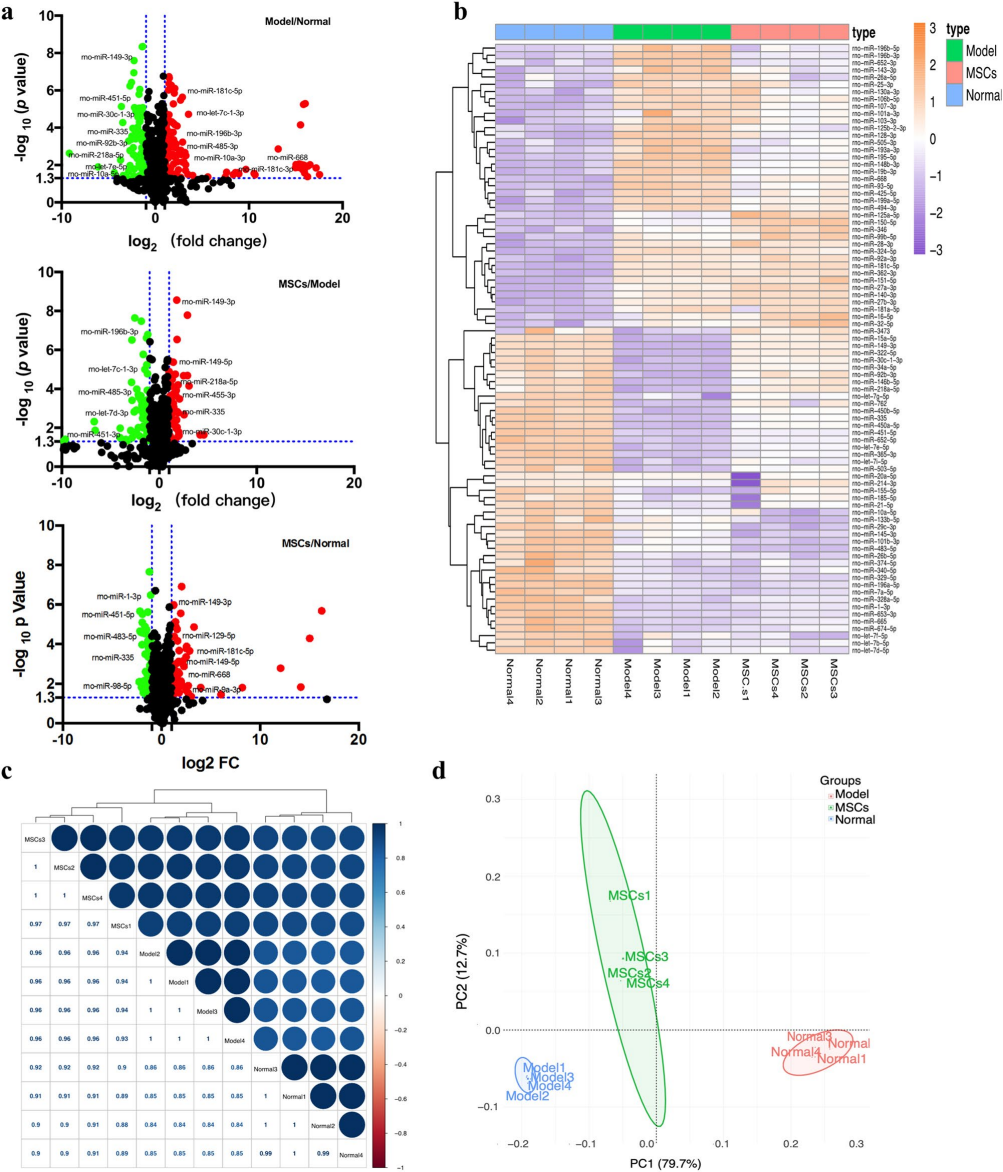
Analysis of miRNA prolifing. (**a**) Volcano plot displaying the differentially expressed miRNA in Model/Normal, MSCs/Model, and MSCs/Normal groups by applying a twofold change expression difference with *p* < 0.05. (**b**) Heatmap display of normal, model and MSCs groups enriched differentially expressed miRNAs. (**c**) Hierarchical clustering analysis of miRNAs based on the correlation matrix among the normal, model and MSCs groups. (**d**) PCA of differentially expressed miRNAs profiling data to separate the whole samples into three groups.

Uterine tissue RNA samples from the animals of normal, model, and MSCs treatment groups were analyzed using an miRNA chip. miRNAs showing two-fold expression differences with statistical significance (*p* < 0.05) between every two groups were screened and analyzed. In total, 539 miRNAs in Model/Normal groups, 529 miRNAs in MSCs/Model groups, and 467 miRNAs in MSCs/Normal groups were analyzed and visualized by volcano plot. The red and green dots represent miRNAs whose expressions were upregulated or downregulated by more than two-fold, respectively. There were 108 and 77 miRNAs with upregulated and downregulated expressions in the model group compared with the normal group; expression levels of 55 miRNAs were upregulated and those of 59 miRNAs were downregulated in the MSCs group compared with the model group (Fig. 3a). With the above differentially expressed miRNAs, 45 miRNAs were found to be downregulated in the model group, upregulated in the MSCs group compared with the model group; and 39 miRNAs were upregulated in the model group, while downregulated in the MSCs group (Fig. 3b). To obtain an overview of the relationships among different treatment groups and samples, a complete dataset was generated using 84 differentially expressed miRNAs through correlation analysis (Fig. 3c), unsupervised HC analysis, and PCA (Fig. 3d). The relationship between each sample obtained by these algorithms was consistent with our experimental results.

### miRNA-mRNA interaction and gene ontology analysis

**Figure 4:**
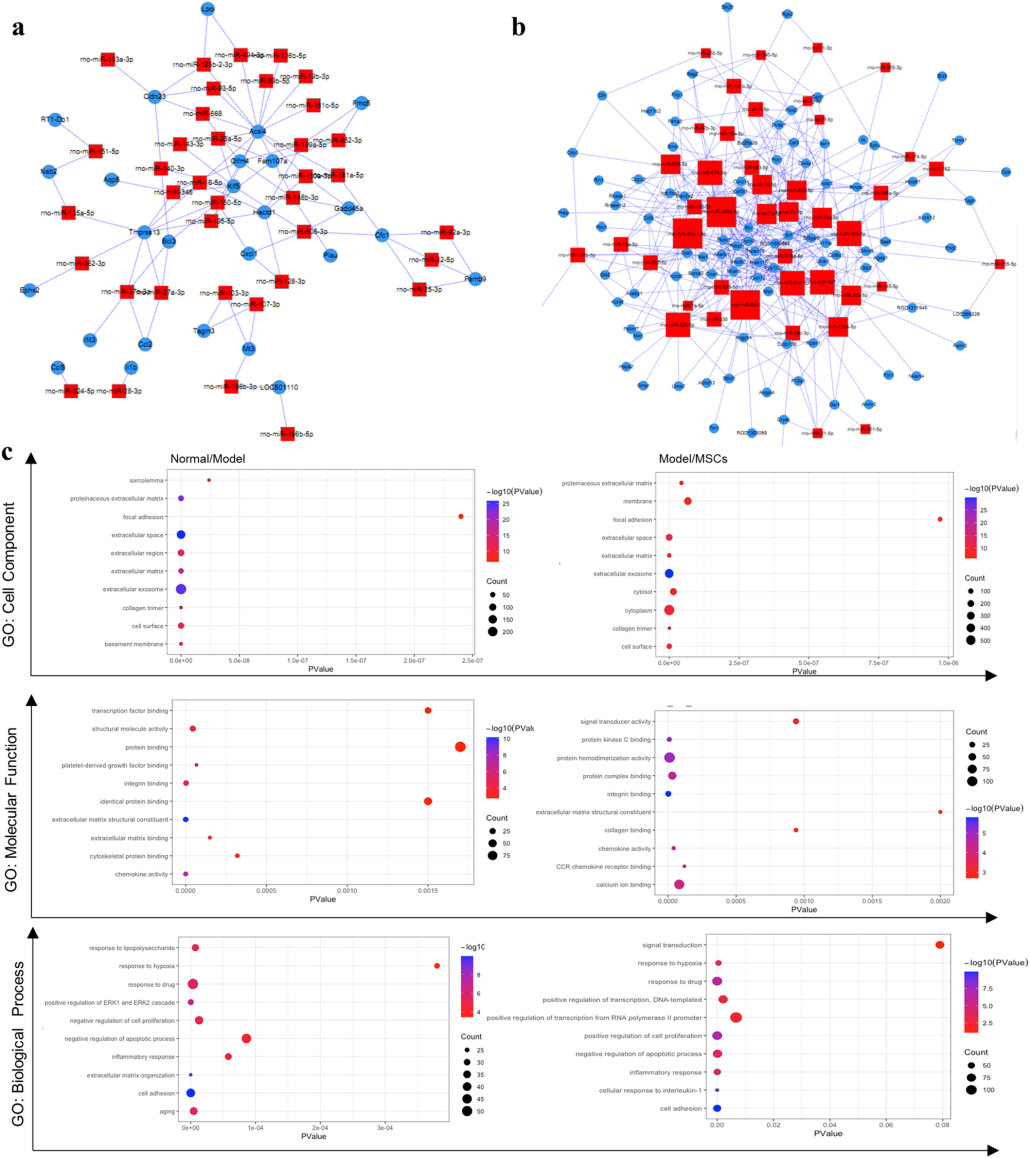
MiRNAs /mRNAs interaction and differently expression gene functional analysis. (**a**) miRNAs up-regulation after injury and down-regulation by UC-MSCs transplantation and their interaction mRNAs. (**b**). miRNAs down-regulation after injury and up-regulation by UC-MSCs transplantation and their interaction mRNAs. (**c**) Representative GO terms (cell component, molecular function and biological process) of the Normal/Model and Model/MSCs enriched mRNAs.

The mRNA array showed that expression levels of 615 mRNAs were downregulated and that of 421 mRNAs were upregulated in the model group compared with the normal group; expression levels of 1192 mRNAs were downregulated and those of 875 mRNAs were upregulated in the MSCs group compared with the model group. Similarly, expression levels of 1027 mRNAs were downregulated and those of 591 mRNAs were upregulated in the MSCs group compared to the normal group (Additional file 1: Figure S2a–b). Among these genes and differentially expressed miRNAs, two miRNA- mRNA interaction networks were created to show that certain miRNAs were upregulated in the model group, but downregulated in the MSCs group and the mRNAs interacting with it (Fig. 4a) and miRNA downregulated in the model group, upregulated in the MSCs group, and the mRNAs interacting (Fig. 4b).

GO enrichment analysis provides a hierarchically organized set of thousands of standardized terms for cell components, molecular functions, and biological pathways. Figure 4c shows the differentially expressed genes related to GO terms in the model group compared with the normal group (Normal/Model) and in the MSCs group compared with the model group (Model/MSCs). Cell components showed that the ECM and extracellular exosome are involved in the processes of uterine injury and repair. Molecular function showed that structural molecular activity, protein activity, and protein binding were enriched in uterine injury and repair processes. Biological process terms showed that cell proliferation and apoptosis regulation, inflammatory response, and cell adhesion were enriched in the processes of endometrial injury and repair.

### Pathway analysis

**Figure 5:**
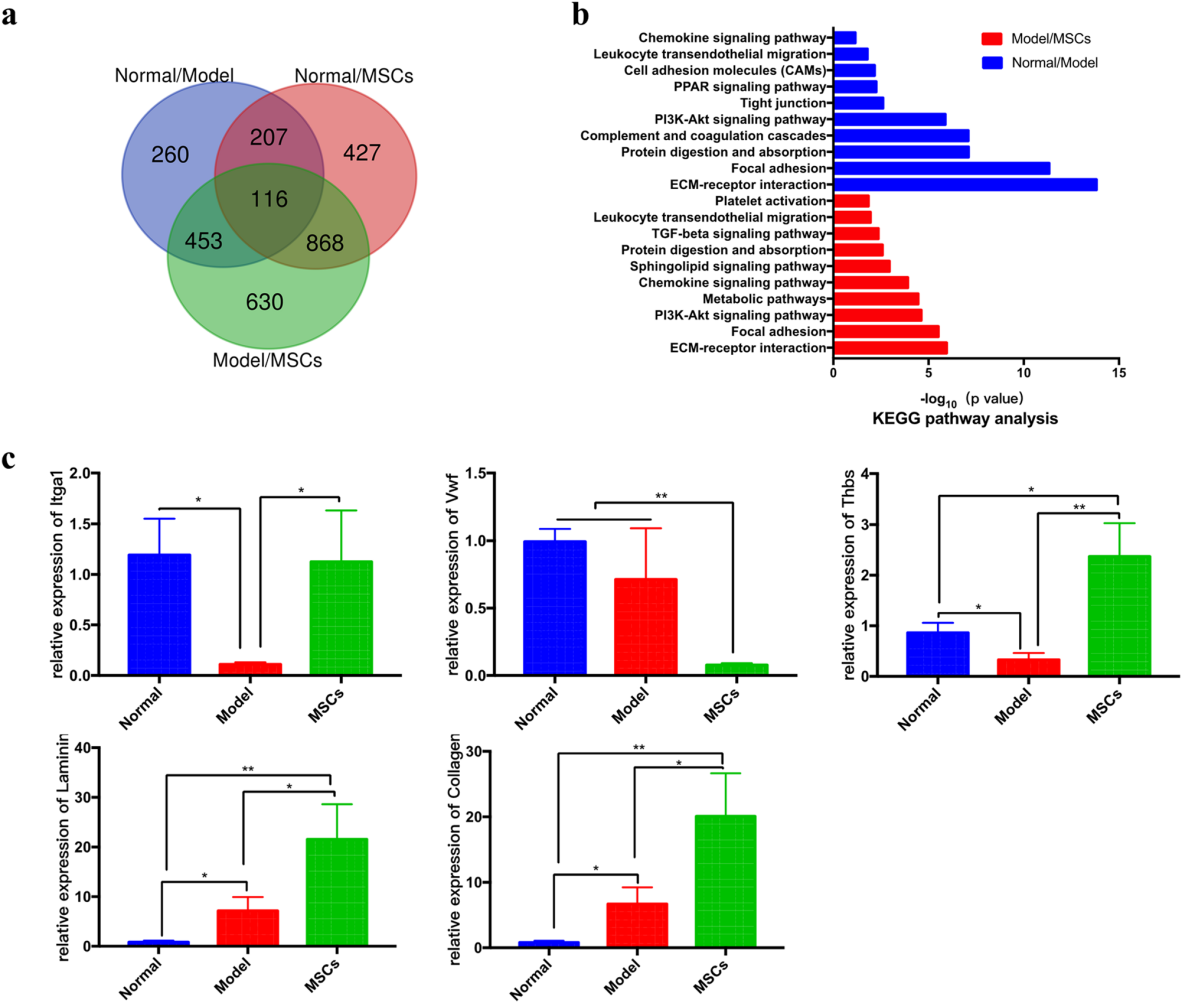
Relationship and functional analysis of differentially expressed mRNAs. (**a**) Venn diagram of genes differentially expressed in Normal/Model, Model/MSCs and Normal/MSCs groups. (**b**) KEGG pathway analysis^36^ of Normal/Model and Model/MSCs groups measured by mRNA microarray chip. (**c**) qRT-PCR verification of differentially expressed genes in ECM-receptor interaction signal pathway (These genes were obtained from MAP04512 and MAP04510 of KEGG: https://www.kegg.jp/kegg/). *p < 0.05, **p < 0.01.

To obtain a global view of endometrial injury and UC-MSCs treatment in rats, we selected 1036 differentially expressed mRNAs in Normal/Model, 2067 in Model/MSCs, and 1618 in Normal/MSCs and then mapped their relation by Venn diagram (Fig. 5a). KEGG pathway analysis was divided into two parts according to endometrial injury and UC-MSCs transplantation repair. The majority of the genes that were differentially expressed in the Normal/Model groups were enriched in several pathways, such as ECM-receptor interaction, focal adhesion, and protein digestion and absorption. The Model/MSCs groups mainly utilized ECM-receptor interaction, focal adhesion, PI3K-Akt and chemokine signaling pathway. Overall, the KEGG pathways involved in uterine injury and UC-MSCs therapy were consistent with the GO terms (Fig. 5b). Collectively, these findings suggest that the destruction of the ECM, which is responsible for maintaining the structure and function of cells and tissues, and the degradation and absorption of proteins drive the uterine injury process. UC-MSCs transplantation mainly plays a regulatory role in the chemokines and inflammatory response, as well as in the ECM reconstruction. The results of qRT-PCR showed that the expression of some key genes involved in ECM-receptor interaction changed dynamically in different groups, *Itga1* and *Thbs* expression decreased in the model group and increased in MSCs group, *Laminin* and *Collagen* expression increased in both the model group and MSCs group, and *Vwf* decreased in MSCs group compared with normal and model control, indicating their broader roles in the process of endometrial injury and repair (Fig. 5c).

## Discussion

The endometrium is a highly regenerative tissue that undergoes more than 400 cycles of formation, development, and shedding during a woman's reproductive age. Inflammation, endometrial dysfunction, and frequent uterine surgeries result in a thin endometrium. To reveal the mechanisms underlying endometrial injury and repair, this study used the rat model of endometrial injury and gene microarray chip platform to analyze the effects of UC-MSCs on the rat endometrium in terms of histomorphology and repair of the damaged microenvironment. Use of 95% ethanol perfusion in rats to induce endometrial injury adopted in this study is a well-established method and offers advantages including simple modeling operations, high success rates, and stable phenotypes. In vivo experiments showed that UC-MSCs exert good reparative effects on the morphology and function of injured rat uteri. Qi’s results showed that inhibition of cell viability by mifepristone depended on drug concentration and treatment time^23^. Based on their results, we speculated that mifepristone treatment could approach the clinical endometrial injury environment better, explored the viability of mifepristone treated ESCs, and selected mifepristone dose (20 μM) that significantly inhibited cell viability for follow-up in vitro studies.

Recently, based on in-depth research studies on stem cells, cell-based therapy has shown great promise for the restoration of a thin endometrium^7,16,24^. One of the main benefits of MSCs-based treatments is that they can preferentially home to damaged tissues and organs^25^. Following previous reviews, Mujib takes homing to encompass both non-systemic and systemic homing: In non-systematic homing, MSCs are locally transplanted to the target tissue, and then guided to the injury site through a chemokine gradient. During system homing, MSCs are injected or endogenously recruited into the blood, and then must go through a multi-step process to exit the circulation and migrate to the injury site^26,27^. In our study, UC-MSCs tail vein transplantation belongs to systemic homing via multiple steps: (1) tethering and rolling, (2) activation, (3) arrest, (4) transmigration or diapedesis, and (5) migration^28^. The anti-human nuclear antibody, MAB1281 was used to mark the localization of UC-MSCs in the endometrium^29^. The MAB1281 immunohistochemical analysis and transwell 3D experiments revealed the homing of UC-MSCs to injured endometrial cells.

The process of endometrial regeneration and differentiation requires the transition of cells between mesenchymal and epithelial phenotypes, known as mesenchymal-epithelial transition (MET) and epithelial-mesenchymal transition (EMT)^30^. According to McCormack’s^22^ research, the separation of ESCs may mix part of the epithelial cells and myometrial cells. Hence, we used an anti-vimentin antibody to detect the purity of ESCs. Studies have reported that some cancer-derived epithelial cells express vimentin, but normal epithelial cells are keratin skeletons and do not express Vimentin. Vimentin expression begins to appear during the EMT^31–33^. We did not perform flow cytometric identification of ESCs due to the following reasons: First, Stromal cells began to adhere 0.5 h after inoculation, while most gland cells adhered 24 h after inoculation. We changed the medium after 6 h of inoculation to remove the non-adherent glandular cells, which can ensure that most of the obtained were stromal cells. Second, studies revealed that immunohistochemical staining analysis using normal uterine tissue sections showed that epithelial cells hardly express vimentin^21,34^, and our immunocytochemical staining results show that ESCs have a high purity. Third, studies have reported that a variety of chemokines, adhesion factors, growth factors and other signaling molecules are expressed locally in damaged tissue, attracting UC-MSCs homing^25^. Although the isolated ESCs may have been mixed with epithelial cells or glandular cells, it would be more like in vivo injury environment. Thus, ESCs obtained in our study were suitable for evaluating UC-MSCs homing.

The current study presents an overview of differentially expressed miRNAs and mRNAs during endometrial injury and repair processes. The putative mRNA targets of miRNAs that were analyzed and identified by mRNA microarray and the Targetscan website, and miRNA-mRNA interaction networks were mapped to analyze the changes in the microenvironment during endometrial injury and repair. The KEGG pathway annotation^35,36^ showed that ECM-receptor interaction, focal adhesion, and PI3K-Akt signaling pathway displayed significant changes in the processes of endometrial injury and repair in rats. The ECM signaling pathway was shown to played an important role in morphogenesis of tissues and organs as well as the maintenance of cell and tissue structure. The dynamic characteristics of the ECM play key roles in development, wound healing, and certain pathological states^37,38^. The ECM can interact with integrins to transmit a variety of signals to regulate key cellular processes, such as cell proliferation and migration^39^. The focal adhesions formed by ECM-integrin-cytoskeleton proteins are the structural basis of integrin signal transduction. Many signal proteins combine with focal adhesions to play a role in the signal transduction of integrin^40–43^. Integrin alpha (*ITGA*) was identified as hub genes in network analysis and predicted to be involved in binding laminin and collagen, which played key roles in ECM-receptor interaction and focal adhesion pathways^44,45^. thrombospondin-2 (*THBS2*), as an extracellular matrix protein encoding gene, can encode a secreted ECM glycoprotein, which has a certain correlation with the severity of tissue fibrosis^46,47^. Von Willebrand factor (*VWF*) promoted shear stress-regulated platelet aggregation and platelet clearance, mediated platelet adhesion to damaged blood vessels and stabilized blood coagulation factor^48,49^. The results reported in earlier studies are consistent with the findings of the present study, based on these results, severe structural tissue damage and protein degradation occurs after endometrial injury, resulting in damaged cells and tissues releasing a variety of cytokines. The body in the inflammatory state is unable to repair the damaged tissue completely. After UC-MSCs tail vein transplantation, several cytokines induced UC-MSCs homing to the injured site, regulated inflammation, restored blood supply, and stimulated tissue repair through paracrine effects, instead of simply replacing the damaged ESCs. Though microenvironment changes in the endometrium have been revealed, elucidation of the underlying mechanisms requires further investigations. Furthermore, whether these interacting miRNAs and mRNAs could improve the restoration of damaged tissues by UC-MSCs remains to be explored.

For the first time, we have identified two miRNA-mRNA networks associated with thin endometrial injury and UC-MSCs transplantation repair. Bioinformatics analysis and prediction, and qRT-PCR verification were performed to explore the mechanisms underlying UC-MSCs-induced endometrial repair, but further studies are warranted to validate our data. In summary, our research uncovered the reparative effects of UC-MSCs on thin, injured endometrium in rats, and provides a theoretical basis for future research in this field.

## Conclusion

In this study, we found that UC-MSCs migrate to the damaged endometrium and promote the recovery of thin endometrium and its structure and function. In vitro studies have shown that UC-MSCs migrate to injured ESCs and enhance their proliferative capacity. Our study revealed the relationship between miRNAs and mRNAs during endometrial injury and repair processes. We believe that our study outcomes provide new avenues for exploring the reparative effects of UC-MSCs on injured endometrium.

## Supplementary Information

Supplementary Information 1.

Supplementary Information 2.

## Data Availability

All data generated or analyzed during this study are included in this published article and supplementary material.

## Abbreviations

UC-MSCs: Umbilical cord mesenchymal stem cells
IUA: Intrauterine adhesions
GO: Gene ontology
KEGG: Kyoto encyclopedia of genes and genomes
qRT-PCR: Quantitative reverse-transcription polymerase chain reaction
ESCs: Endometrial stromal cells
ECM: Extracellular matrix
MSCs: Mesenchymal stem cells
BMSCs: Bone marrow-derived mesenchymal stem cells
FACS: Fluorescence-activated cell sorting
H&E: Hematoxylin–eosin staining
PCA: Principal component analysis
HC: Hierarchical clustering analysis
