# Supplementary material for: Transplantation of umbilical cord-derived mesenchymal stem cells promotes the recovery of thin endometrium in rats
Source: Sci Rep. 2022 Jan 10;12:412. doi: 10.1038/s41598-021-04454-7 (PMC8748676; doi:10.1038/s41598-021-04454-7)
Supplement: Supplementary file 1 — Supplementary Information 1. [file 41598_2021_4454_MOESM1_ESM.docx]

**Supplementary materials**


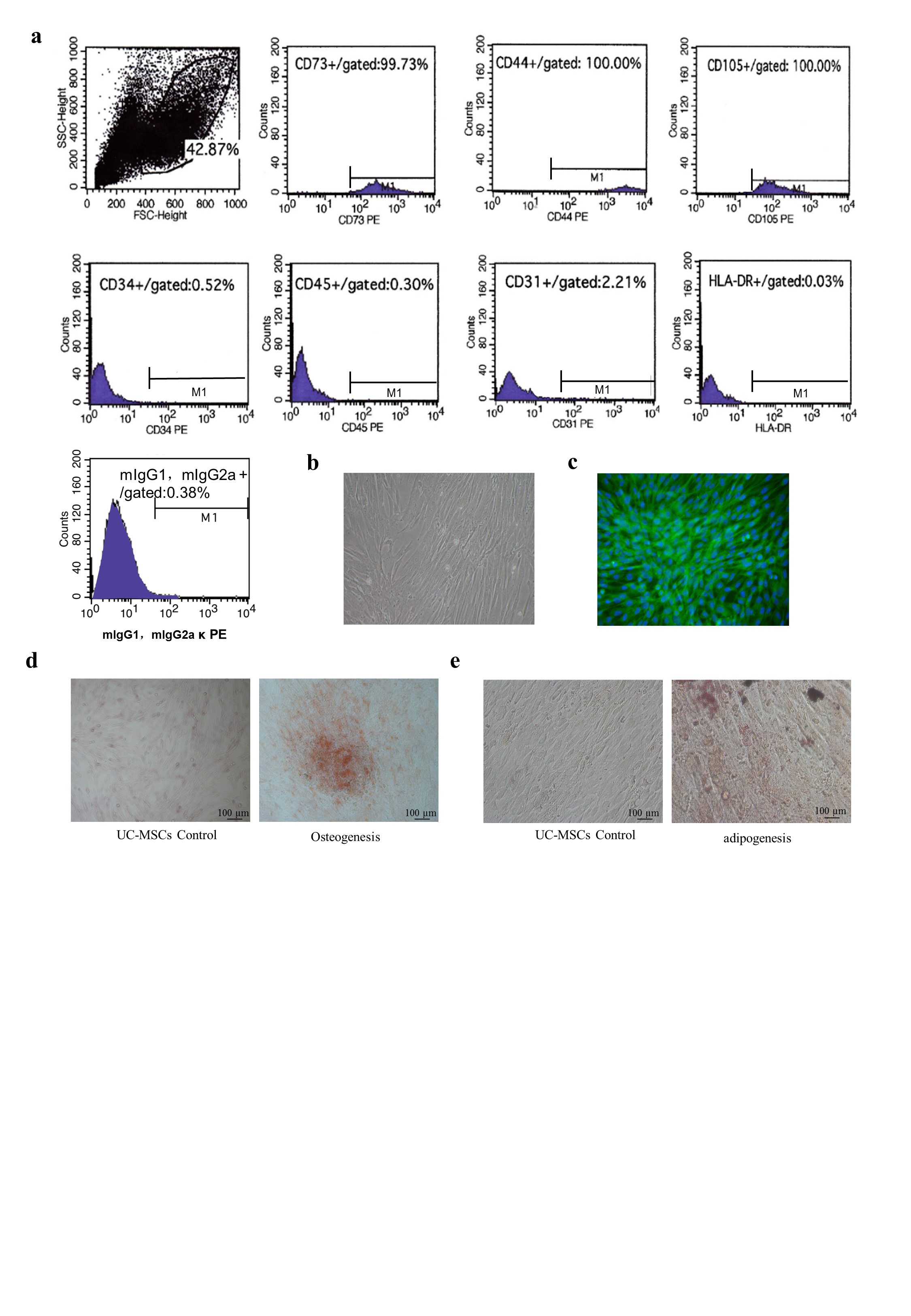


**Figure S1** Identification of human umbilical cord mesenchymal stem cells. **a**. Staining of CD73, CD44, CD105, CD34, CD45, CD31, HLA-DR and PE hMSC Negative Isotype Control analyzed by Flow cytometry. **b**. UC-MSCs observation under discrepancy microscopy at F5 culture. **c**. CD44 immunofluorescence identification of UC-MSCs. **d**. Normal and osteogenesis UC-MSCs was confirmed by Alizarin red staining to show calcium deposition (200×). **e**. Normal and adipogenesis UC-MSCs was confirmed by Oil red O staining to show lipid accumulation (200×).


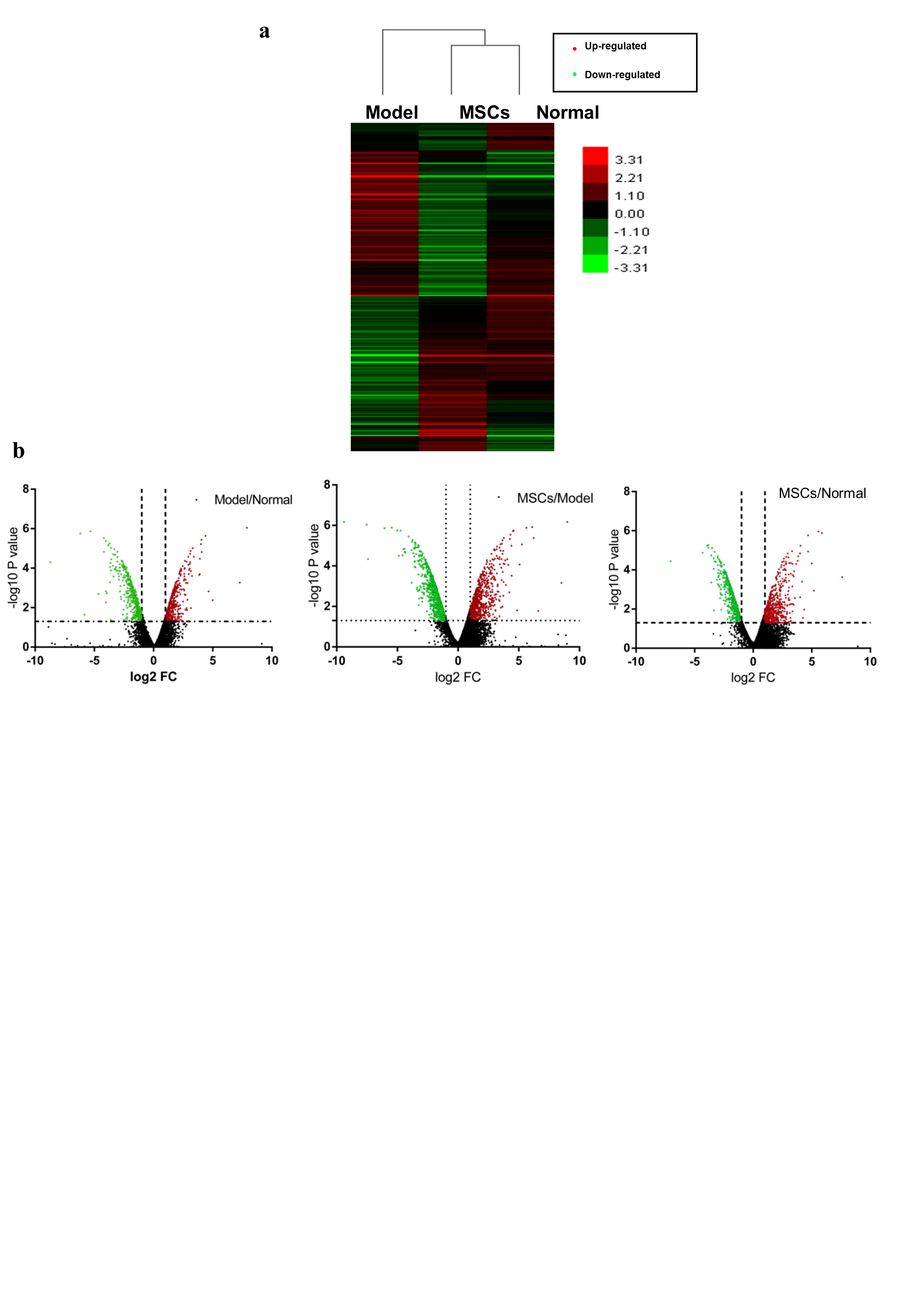


**Figure S2** Analysis of mRNA prolifing. **a**. Heatmap display of normal, model and MSCs groups enriched differentially expressed mRNAs. **b**. Volcano plot displaying the differentially expressed mRNA in Model/Normal, MSCs/Model, and MSCs/Normal groups by applying a twofold change expression difference with p < 0.05.
